# Psychological characteristics and emotional difficulties underlying school refusal in adolescents using functional near-infrared spectroscopy

**DOI:** 10.1186/s12888-023-05291-w

**Published:** 2023-12-01

**Authors:** Gaizhi Li, Ying Niu, Xiumei Liang, Elissar Andari, Zhifen Liu, Ke-Rang Zhang

**Affiliations:** 1 Department of Psychiatry, First Hospital of Shanxi Medical University, No 85 Jiefang Nan Road, Taiyuan, 030001 Shanxi Province China; 2 First Clinical Medical College, Shanxi Medical University, Taiyuan, Shanxi Province China; 3 College of Medical Sciences, Shanxi Medical University, Taiyuan, Shanxi Province China; 4 Department of Psychiatry, College of Medicine and Life Sciences, University of Toledo, Toledo, OH USA

**Keywords:** School refusal, Adolescent, fNIRS

## Abstract

**Background:**

This study aims to explore the psychological characteristics, related emotional problems and potential NIR brain function mechanism of adolescents who refuse to attend school.

**Methods:**

The study included 38 adolescents (12–18 years old) who were not attending school and 35 healthy controls (12–18 years old) who are attending school regularly. Participants completed (1) general demographics, (2) Eysenck Personality Questionnaire (EPQ), (3) Zung Self-Rating Depression Scale (SDS), (4) Zung Self-Rating Anxiety Scale (SAS), and (5) Symptom Checklist-90 (SCL-90). In addition to the clinical tests, participants completed functional near-infrared spectroscopy (fNIRS). Mental health, personality, and emotional state were evaluated in both groups to explore the differences and to understand the underlying mechanisms of school refusal during adolescence.

**Results:**

Adolescents who did not attend school had higher neuroticism scores on the Eysenck Personality Questionnaire than healthy controls (*p(FDR)* < 0.001), introversion and concealment scores were lower than those of healthy controls (*p(FDR)* < 0.001), there was no significant difference in psychoticism scores between groups. SDS, SAS, SCL-90 scores and factor scores were higher than those of healthy control group (*p(FDR)* < 0.001), NIR functional brain imaging was different from healthy control group in the 12 and 27 channels (*p(FDR)* = 0.030, *p(FDR)* = 0.018), and no difference was found in the remaining channels (*p(FDR) >* 0.05). There were statistically significant differences in age and gender between the adolescents who refused school and the control group (*p(FDR)* < 0.001).

**Conclusion:**

School refusal adolescents are relatively introverted and sensitive and need more attention in daily life. Although the adolescents’ emotional problems did not reach the diagnostic criteria of depressive disorder and anxiety disorder, their scores were still higher than those of the control group, suggesting that we should pay more attention to their emotional problems in order to better help them return to school. Using fNIRS, it was found that abnormalities in frontal lobe regions in adolescents with school refusal behaviors, which would contribute to early diagnosis and timely intervention of school refusal behaviors.

## Introduction

School refusal is a common emotional problem in teenagers. School refusal (SR) refers to the spontaneous reluctance of the child or adolescent to attend school and/or the difficulty in staying at school for the entire day with the knowledge of the parent [1, 2]. Studies have shown the incidence of school refusal in school-aged children varies from 1-5% [3]. There are no known gender differences in SR [4]. However, studies have shown that school refusal is more common in two age groups : children aged between 5 and 7 years old and adolescents aged between 11 and 14 years old [5]. It’s reported that individual factors, family factors and school factors are closely related to adolescents’ school refusal behavior [6–8]. Previous studies have shown that extraversion, neuroticism and psychoticism are related to school refusal: extroverted adolescents who fail to meet academic requirements may turn their interest to other things besides learning in order to pursue happiness, resulting in school refusal [9, 10]. Highly neuroticism adolescents have a higher prevalence of school refusal because they are emotionally unstable and are often accompanied by a sense of victimization [8]; Highly psychoticism adolescents show maladjustment nature. These adolescents lack of care for others and strained interpersonal relationships with classmates, which increases the prevalence of school refusal [9]. Inhibited and self-critical personality traits have also been found with school refusal, which is characterized by feeling of shame and self-critical tendencies in social situations. Adolescents with these personalities choose school refusal to avoid social situations that might lead to feelings of shame and devaluation [8]. Mental health problems such as anxiety, depression and oppositional defiant disorder were also related to school refusal [8, 11], for example, adolescents with separation anxiety disorders choose school refusal to draw attention to significant others; school refusal individuals for out-of-school tangible reinforcement often have oppositional defiant disorder [12].Many adolescents suffer from academic burnout due to academic pressures which could be one of the leading causes to SR [13]. SR and truancy have direct short-term consequences which include academic failure, isolation by peer groups, deterioration of parent-child relationship, and violence or delinquency [14, 15]. SR is also associated with long-term negative consequences on the development of socialization, education, and on increasing risk for substance abuse, marital crisis, employment difficulties during adulthood and mental disorders [14–16].

Cultural differences in school refusal behavior have been reported [17]. In China, the prevalence of school refusal is increasing year by year, most studies have focused on the definition of school refusal and some influencing factors [18]. Liu et al. suggest that the development and maintenance of school refusal behavior in Chinese adolescents is the result of the interaction between the social environment, family conflict, and individual psychological factors. There are five main aspects: (1) a competition-oriented social environment; (2) a conflict-ridden family living space; (3) a lack of supportive personal living space; (4) a conflict between the pros and cons of being labeled as psychiatric diagnosis; (5) reintegrating into school life [19]. While little attention has been paid to the personality traits, emotional and neural underpinnings of Chinese adolescents’ school refusal behavior. Xu et al. suggest that the development of school refusal is a process from cognition to emotion to externalized behavior and is gradually serious [20]. Therefore, the study of the emotional manifestations and physiological mechanisms of adolescents who have school refusal can provide a basis for early intervention. Focusing on the emotional characteristics of Chinese adolescents who have school-refusal and disseminating this mental health knowledge to schools and parents can effectively improve the early identification of school refusal behavior, support and guide adolescents to seek help from professionals, and reduce the risk of adolescents dropping out of school. No studies about the mechanism of SR is reported until now. Studying the neural underlying mechanisms of school refusal will lead to more accurate early diagnosis of this behavior, which provide early identification and individualized treatment.

Here, in this study, we aim to study the emotional mechanisms and brain correlates of the emotional difficulties that underly school refusal during adolescence, using functional near-infrared spectroscopy (fNIRS). As previous studies reported the emotional problems of adolescents with school refusal [7, 15, 21], in addition, the frontal and temporal cortex is correlated with the emotion and cognition [22–25]. fNIRS is a new non-invasive technique that is capable of measuring changes in oxygenated and deoxygenated hemoglobin of frontal and temporal cortex. fNIRS has advantages such as low cost, harmless to participants, easy to use, and well endured [26],and has been widely used in the study of mental disorders, such as depressive disorder, bipolar disorder, schizophrenia and others [27]. The verbal fluency test (VFT) is most often used in the collection of fNIRS data. fNIRS in conjunction with the VFT has been widely used in psychiatric research. In various mental disorders, frontal and temporal lobe regions of the brain are significantly less activated (i.e., the increase in Hbo is significantly reduced) during the VFT [28]. For example, in patients with schizophrenia, it has been shown that using the VFT during fNIRS testing reduces the increase in frontal and temporal lobe Hbo in patients compared to controls, implying that patients with schizophrenia experience hemodynamic changes [28, 29]. Depressed patients have reduced levels of left prefrontal activation during the VFT and poor task performance [30]. The VFT strategically accesses lexical-semantic information, so there is a reliance on and activation of the superior medial frontal cortex, the ventral lateral prefrontal cortex (VLPFC), and the anterior temporal lobes during the VFT, especially the left hemisphere [28, 31–33].

However, at present, no studies have used fNIRS and VFT to explore the mechanism of brain function in adolescents who refuse to go to school. Therefore, in addition to exploring the psychological characteristics and emotional difficulties of adolescents with SR, we are using fNIRS in combination with VFT to explore differences in brain function between adolescents who refuse school and control adolescents who do not have trouble with school refusal, particularly in the frontal and temporal lobe regions of school refusal in adolescents.

## Methods

### Participants

In this study, 38 adolescents (aged between 12 and 18 years old) were recruited in the study between February and December 2019. Recruitment was based on patients’ admission to the Children and Adolescents Outpatient Department of Mental Health, the First Hospital of Shanxi Medical University. The inclusion criteria consisted of: (1) age between 12 and 18 years old; (2) functional assessment of school refusal as suggested by Kearney and Albano,2004 [34]; (3) more than 50% of school absence or absence in the 4 weeks prior to the visit. Exclusion criteria consists of an anxiety and depression diagnosis which is assessed by the psychiatrist of the study using the Mini-International Neuropsychiatric Interview (M.I.N.I.)(compatible with the Diagnostic and Statistical Manual of Mental Disorders, fifth Edition (DSM-5)) [35]. We recruited healthy controls (n = 35) locally from advertisement. The inclusion for the HC including aged 12–18 years old, without gender limitation, the flyers for including healthy controls (aged 12–18 years old, without gender limitation, Han nationality) were posted in middle schools in Taiyuan City. All the informed consent were obtained from the participants themselves and their parents/caregivers. (NO. of ethics approval: KYLL-2023-080).

### Measures

#### General demographic data

We collected demographic data from all participants that included age, gender, and education level.

#### Eysenck Personality Questionnaire

We used the Chinese version of Eysenck Personality Questionnaire (EPQ) that consists of four subscales: extraversion (E), neuroticism (N), psychoticism (P) and lying (L) [36]. Binary answers were provided. The Chinese version of Eysenck Personality Questionnaire has high reliability and validity [37, 38].

#### Zung Self-rating Depression Scale

The Chinese version of Zung Self-rating Depression Scale (SDS) [39] is used for assessment of depressive symptoms which includes 20 items and each item is graded 1 to 4. Some items 2, 5, 6, 11, 12, 14, 16, 17, 18 and 20 are graded in reverse. Previous studies have found the scale to be appropriate and commonly used by Chinese people [40–42]. Mild depression is considered when the total score is between 50 and 59. A moderate depression is considered when the total score is between 60 and 69, and a severe depression is associated to a score above 69. The Chinese version of Zung Self-rating Depression Scale has high reliability and validity [43, 44].

#### Zung Self-Rating anxiety scale

There are 20 items in the Chinese version of Zung Self-Rating Anxiety Scale (SAS) [45], and each item is graded on 1 to 4 levels. Among them, items 5, 9, 13, 17 and 19 are graded in reverse. Studies demonstrate that the scale can be widely used to screen for anxiety in the Chinese population [40, 42, 46]. A score between 50 and 59 is associated with mild anxiety, a score between 60 and 69 is associated with moderate anxiety, and a score above 69 scores is associated with severe anxiety. The Cronbach’s α coefficient was 0.913, and has high constructive validity [47] .

#### Symptom Checklist 90

We used the Chinese version of Symptom Checklist 90 (SCL-90),which consists of 90 items with each item graded 1 to 5. SCL-90 includes 10 factors that reflect somatization, obsessive symptoms, interpersonal sensitivity, depression, anxiety, hostility, phobic anxiety, paranoid ideation, psychoticism, and the other aspects of psychological symptoms(measurement of individual sleep and diet) [48]. If the total score is over 160, or the number of positive items is more than 43, or the mean of factor score is ≥ 2, this will be an indication of mild or above psychological problems. If the mean of each factor score is ≥ 2.5, this indicates that the psychological pain has reached a moderate level or above. If the individual factor score is ≥ 3, this indicates that the pain level has reached a mild or above severe level, indicating the possibility of psychological problems. The Chinese version of SCL-90 has high reliability and validity. The reliability of the general scale was 0.97, and was over 0.67 for all the subscales. Test-retested correlation was over 0.70. SCL-90 had high content validity and constructive validity [49].

### Data collection

The hemodynamic responses in the prefrontal cortices and superior temporal cortices was measured by a 52-channel fNIRS system (ETG-4100. Hitachi Medical Co., Tokyo, Japan) with 2 NIR light wavelengths (695 and 830 nm). The fNIRS system contains 16 light detectors and 17 light emitters, all of which were arranged in a 3 × 11 array to form 52 measurement channels. All the participants were asked to perform a Verbal Fluency Task (VFT) in a quiet environment. Participants were asked to seat with eyes open, avoiding excessive body and head movements, and focusing on a cross on the screen. The VFT test comprised a 30-s pre-task period, a 60-s task period, and a 70-s post-task period. During the pre- and post-task periods, the participants were asked to constantly say “one, two, three, four, five” repeatedly. During the task period, the participants were asked to think as many four-character idioms or phrases as possible, which begin with big, white, and sky [29].

### fNIRS analysis

#### Data preprocessing

The near-infrared spectroscopy signals were preprocessed using the NIRS-SPM toolbox, which is a MATLAB-based software package (MATLAB 2013b). The preprocessing steps included: transforming all .csv files into NIRS-SPM available .mat files; checking for participants’ available channels.

#### Calculate the β-value

The NIRS-SPM toolbox mainly uses the general linear model (GLM) method in data analysis, The GLM is formulated as follows: Y = βX + ε. In this study, β is represents the level of cortical activation during the VFT.

First, low-frequency drift generated by breathing, heartbeat, or other factors was conducted using the discrete cosine transform (DCT). Physiological noise was filtered using a low-pass filter that is based on the hemodynamic response function (HRF).

Second, a GLM was constructed using the time series associated with rest and task performance as the independent variables, the oxyhemoglobin concentration as the dependent variables. The first-order derivative and second-order derivative of the time series were used as covariates in this process.

Third, the value of β was calculated.

#### Index extraction

The values of β were extracted for 52 channels for participants. The degree of activation of the brain cortex during the VFT task was assessed by δβ value of oxy-hemoglobin (VFT β value minus baseline β value).

### Statistical analysis

SPSS 22.0 was used for the data analysis (SPSS Inc, Chicago, IL, USA).

The categorical data (gender) were analyzed with the chi-square test. The numerical data was analyzed using two independent sample test, including age, educational age, duration of SR, total scores and each subscale scores of EPQ, SAS, SDS, SCI-90 (with age, gender and educational years as covariates). A one-way ANOVA was used, with group as the between-group factor (SR group and HC group), and age and gender as covariates. This was used to compare the differences in frontal and temporal cortex activation levels between the SR group and HC group. The p-value was corrected by false discovery rate correction(FDR, the FDR correction method ranks multiple hypotheses according to the magnitude of the p-value and then the significance level of each hypothesis is determined according to the ranked order) [50, 51]. And less than 0.05 was considered statistically significant. Bonferroni test was used for post-hoc analysis to identify the sources of differences (Bonferroni corrections to minimize type I errors, specifically raw p value*number of t tests. Values less than 0.05 reached by the Bonferroni correction were considered statistically significant).

Mean δβ values were extracted for channels with statistically significant results, and correlation between δβ values and clinical symptoms (EPQ subscale scores, SAS total score, SDS total score, and SCL-90 total score) using Pearson’s correlation analysis (with age, gender and educational years as covariates). The p-value was also corrected by FDR.

## Results

### General demographic data on adolescents who refuse to attend school

Age and gender were significantly different between the SR group and HC group (*p* < .001, see Table 1). No significant differences were found between the two groups in years of education (*p* = .677, see Table 1). The average duration of refusal was 10.39 ± 13.29 months for the SR group.

**Table 1:** Comparison of Demographic data, EPQ and emotional problems in the SR and HC

Measures	SR Group (n = 38)	HC Group (n = 35)	t/x^2^	*p*
Demographic data				
Age	14.42 ± 1.52	17.14 ± 0.81	-9.661	< 0.001
Gender (male: female)	22:16	4:31	17.154	< 0.001
Educational years	9.27 ± 1.42	9.47 ± 1.83	-0.419	0.677
Duration of SR (months)	10.39 ± 13.29	-	-	-
EPQ				
EPQ-E	31.07 ± 12.32	55.86 ± 10.74	-8.857	< 0.001
EPQ-N	63.31 ± 11.29	48.57 ± 13.59	4.893	< 0.001
EPQ-L	41.22 ± 7.63	48.93 ± 8.21	-4.003	< 0.001
EPQ-P	49.04 ± 9.48	53.57 ± 13.37	-1.603	0.114
SDS total score	64.83 ± 12.94	50.64 ± 11.75	4.866	< 0.001
SAS total score	52.36 ± 9.75	43.86 ± 11.07	3.462	< 0.001
SCL-90				
SCL-90 total score	221.00 ± 62.42	131.26 ± 52.42	6.567	< 0.001
SCL-90 general severity index	2.47 ± 0.69	1.46 ± 0.58	6.648	< 0.001
SCL-90 somatization	1.79 ± 0.69	1.31 ± 0.59	3.049	< 0.001
SCL-90 obsessive-compulsive symptoms	2.81 ± 0.88	1.79 ± 0.69	5.309	< 0.001
SCL-90 interpersonal sensitivity	2.75 ± 0.89	1.62 ± 0.78	5.545	< 0.001
SCL-90 depression	2.88 ± 1.05	1.49 ± 0.68	6.435	< 0.001
SCL-90 anxiety	2.28 ± 0.77	1.39 ± 0.64	5.231	< 0.001
SCL-90 hostility	2.68 ± 0.99	1.49 ± 0.61	5.795	< 0.001
SCL-90phobic anxiety	2.26 ± 0.82	1.49 ± 0.61	4.408	< 0.001
SCL-90 paranoid ideation	2.49 ± 0.82	1.39 ± 0.62	6.191	< 0.001
SCL-90 psychoticism	2.22 ± 0.76	1.35 ± 0.50	5.492	< 0.001
SCL-90 other factor (measurement of individual sleep and diet)	2.15 ± 0.69	1.38 ± 0.56	5.045	< 0.001

*SR* School refusal, *HC* Healthy controls, *EPQ* Eysenck Personality Questionnaire, *SDS* Zung Self-rating Depression Scale, *SAS* Zung Self-Rating Anxiety Scale, *SCL-90* Symptom Checklist 90

### Personality characteristics of SR adolescents

**Fig. 1:**
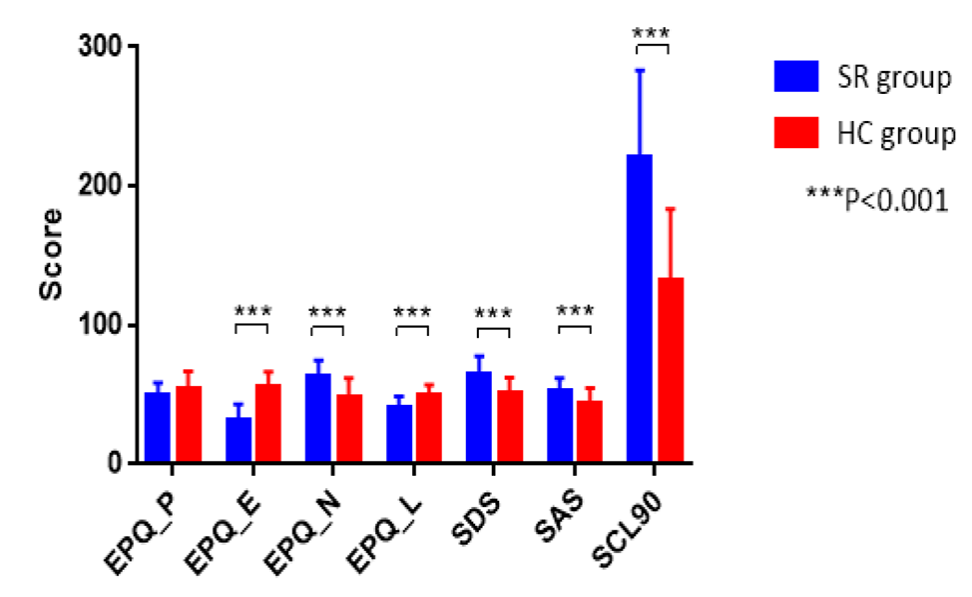
The difference of EPQ, SDS, SAS and SCL-90 between the two groups

It was found that the school refusal group had lower scores of extraversion (E) and concealment (L), and higher scores of neuroticism (N) than the control group (*p* < .001), significant after Bonferroni corrections or multiple comparison (4 subscales). There were no significant differences in psychoticism (P) score between the two groups (*p* = .114, the p-value was corrected using FDR). The results remained the same after controlling for age and gender (see Table 1; Fig. 1).

### Emotional characteristics of SR adolescents

The scores of SAS, SDS, SCL-90 of the SR group were higher than the scores of the HC group (*p* < .001, the p-value was corrected using FDR). SDS scores of the SR group were in the range of moderate depression. SAS scores were in the range of mild anxiety, and scores of other factors except somatization factor were all higher than 2 in the SR group. These results remained the same after controlling for age and gender (see Table 1; Fig. 1).

### Neural correlates of school refusal adolescents

**Fig. 2:**
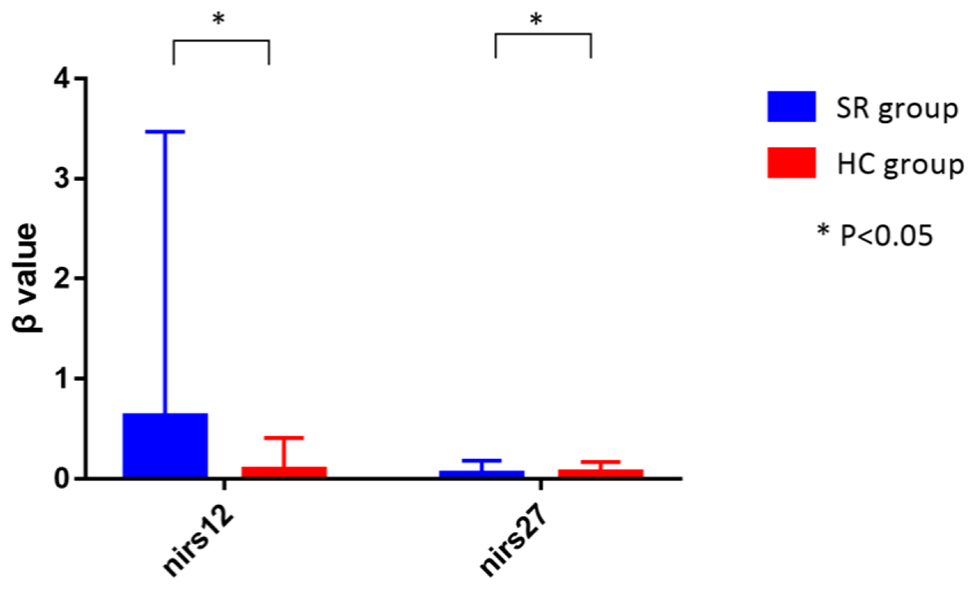
The difference of NIRS Channels between the two groups

When comparing brain activity between the two groups, it was found that the δβ value in channel 12 in the SR group was higher than in HC group (*p* = .030, the p-value was corrected using FDR). The δβ value in channel 27 was found lower in SR group than in HC group (*p* = .018, the p-value was corrected using FDR) (see Table 2; Fig. 2).

**Table 2:** The differentiated channels between the two groups

	SR Group (n = 38)	HC Group (n = 35)	F	*p*
Channel 12	0.626 ± 2.841	0.087 ± 0.321	4.922	0.030
Channel 27	0.049 ± 0.131	0.063 ± 0.105	5.895	0.018

We correlated the brain activity in these channels with the duration of school refusal as well with subscales of EPQ, SAS total score, SDS total score, and SCL-90 total score, with age and gender as covariates. We found only a significant correlation between channel 27 and EPQ-E (r=-.486, *p* = .019, the p-value was corrected using FDR), EPQ-N (r=-.419, *p* = .047, the p-value was corrected using FDR) (See Table 3)and no other correlations were found. We therefore found that activity in frontal areas was negatively correlated with extraversion and neuroticism. Higher scores in neuroticism which can encompass the negative valence in the SR groups is predicted by lower fNIRS activity in frontal areas.

**Table 3:** The correlation of clinical variables and differentiated channels

		EPQ-P	EPQ-E	EPQ-N	EPQ-L	SDS	SAS	SCL-90
Channel 12	r	− 0.152	− 0.089	0.195	0.275	0.298	0.295	0.15
	*p*	0.490	0.686	0.371	0.204	0.168	0.171	0.494
Channel 27	r	− 0.118	− 0.486	− 0.419	0.061	− 0.177	− 0.138	− 0.078
	*p*	0.592	0.019	0.047	0.782	0.420	0.529	0.722

## Discussion

In this study, we studied the behavioral and neural correlates using fNIRS technology of adolescents with school refusal behavior (SR) and healthy controls.

The significant difference between the gender and age of the SR group and the HC group suggests a mismatch between the gender and age of the participants in the two groups in this study, which is a limitation of this study. During the data analysis, we have included gender and age as covariates to control the effect that demographic data to the results. In addition, in this study, the mean of the age of the participants in the SR group was 14.42, which is representative of adolescents with school refusal behaviors, and this stage is in the middle of an individual’s adolescence. In this stage, in addition to great physical changes, the environment (including society, school, and family) of the individual is also changing, and family conflicts increase during adolescence [52], which suggests that problematic family functioning is associated with adolescents’ school refusal behavior [53].

We assessed their emotional states and personality characteristics using EPQ, SDS, SAS, and SCL-90. First, we found that adolescents with SR scored higher on neuroticism subscale of the EPQ than controls. They also scored lower on extraversion subscale of the EPQ than controls. Lower scores on extraversion and higher scores on neuroticism in EPQ have been previously linked to higher mental health problems [54]. Lower scores in extraversion and higher scores in neuroticism are associated with higher risk for depression and anxiety [54, 55]. Our findings are in line with previous studies on school refusal where they found that children and adolescents are more timid, lonely, and withdrawn compared to controls [56].

Previous studies on SR children found that they score higher on psychoticism and neuroticism and scored lower on extraversion compared to controls [56]. These results suggest that adolescents are more likely to have emotion dysregulation and have higher negative states and lower social processing which can impact acceptance to go to school.

Second, adolescents in the school refusal group displayed higher scores of depression and anxiety using SAS and SDS in comparison to healthy controls. This is in line with other studies where they found higher scores of anxiety and depression in school refusal teenagers [13, 14]. Several factors might be influencing adolescents’ school refusal behavior. Family factors are key to adolescents’ school refusal behavior [57]. Interventions with parents as part of a family therapy can significantly alleviate some of the major negative emotions in adolescents with school refusal behavior, such as anxiety, depression, and help them return to school [58].

These studies suggest that we need to pay close attention to the emotional problems of adolescents who refuse to attend school, and take appropriate measures to intervene and encourage them to re-enter school.

Third, at the brain level, our study found statistically significant differences in channels 12 and 27 between the two groups, suggesting that the temporal and frontal oxygenated hemoglobin concentrations in adolescents with SR differ from healthy controls when conducting a cognitive task, which was consistent with the emotional assessment results in this study. Adolescents with SR exhibited lower brain activity in frontal areas (channel 27) during the cognitive task, in comparison to healthy controls. This brain activity in SR group was found negatively correlated with neuroticism (as part of the EPQ personality test). Lower activity in channel 27 was associated with higher scores in neuroticism. School refusal might stem in part from lack of inhibition and less emotion regulation, suggesting that early psychological intervention is needed for school refusal problem.

Our results suggest that there are likely biological underpinnings for school refusal and that fNIRS technology can capture or predict adolescents that are more at risk to refuse to go to school with a lack of activity in frontal areas in response to cognitive tasks. Further studies addressing brain mechanisms in adolescents using NIRS technology might be helpful to better understand the nature of school refusal behavior.

These results also show that adolescents who refuse to go to school have emotional problems that do not yet reach the diagnostic criteria for mental disorders, and that these emotional problems are related to the duration of school refusal. Therefore, it will be helpful to address this problem with psychological consultation that aims to regulate emotions with cognitive treatments. Families should immediately seek professional help and take timely professional intervention measures for adolescents who refuse to go to school, to enhance the chances of adaptive behaviors and to resolve this serious problematic behavior.

### Limitations

One caveats for this study is the relatively small sample size. Future studies with larger sample sizes can explore brain and behavioral differences between the two groups with an increased power. Another limitation is that the age and gender were not matched between groups. Demographic data were added as covariates in our analysis to control for its effects on behavior and brain function. Future studies can conduct follow-up sessions to better explore outcomes.

## Conclusion

Adolescents with school refusal behavior have higher scores in neuroticism and higher depression and anxiety. They also show lower activity in frontal areas during cognitive tasks, measured by fNIRS technology. These results suggest that addressing emotion regulation and enhancing cognitive control early in the process can be necessary to improve prognosis. Future studies with cognitive and family interventions might alleviate some of these symptoms and have kids go back to school and continue their education.

## Data Availability

The data used and analysed during the current study available from the corresponding author, upon on reasonable request.
